# Identification of Reliable Reference Genes for Quantification of MicroRNAs in Serum Samples of Sulfur Mustard-Exposed Veterans

**DOI:** 10.22074/cellj.2015.9

**Published:** 2015-10-07

**Authors:** Sedigheh Gharbi, Mehdi Shamsara, Shahriar Khateri, Mohammad Reza Soroush, Nassim Ghorbanmehr, Mahmood Tavallaei, Mohammad Reza Nourani, Seyed Javad Mowla

**Affiliations:** 1 Department of Molecular Genetics, Faculty of Biological Sciences, Tarbiat Modares University, Tehran, Iran; 2 Department of Biology, Faculty of Sciences, Shahid Bahonar University of Kerman, Kerman, Iran; 3 National Institute of Genetic Engineering and Biotechnology, Tehran, Iran; 4 Janbazan Medical and Engineering Research Center (JMERC), Tehran, Iran; 5 Departmen of Biotechnology, Faculty of Biological Sciences, Alzahra University, Tehran, Iran; 6 Genetic Research Center, Baqiyatallah University of Medical Sciences, Tehran, Iran; 7 Chemical Injury Research Center (CIRC), Baqiyatallah University of Medical Sciences, Tehran, Iran

**Keywords:** MicroRNA, Quantitative Real Time-PCR, Normalization, Sulfur Mustard, miR-423

## Abstract

**Objective:**

In spite of accumulating information about pathological aspects of sulfur mustard (SM), the precise mechanism responsible for its effects is not well understood. Circulating microRNAs (miRNAs) are promising biomarkers for disease diagnosis and prognosis. Accurate normalization using appropriate reference genes, is a critical step in miRNA expression studies. In this study, we aimed to identify appropriate reference gene for microRNA quantification in serum samples of SM victims.

**Materials and Methods:**

In this case and control experimental study, using quantitative real-time polymerase chain reaction (qRT-PCR), we evaluated the suitability of a panel of small RNAs including SNORD38B, SNORD49A, U6, 5S rRNA, miR-423-3p, miR-191, miR-16 and miR-103 in sera of 28 SM-exposed veterans of Iran-Iraq war (1980-1988) and 15 matched control volunteers. Different statistical algorithms including geNorm, Normfinder, best-keeper and comparative delta-quantification cycle (Cq) method were employed to find the least variable reference gene.

**Results:**

miR-423-3p was identified as the most stably expressed reference gene, and miR- 103 and miR-16 ranked after that.

**Conclusion:**

We demonstrate that non-miRNA reference genes have the least stabil- ity in serum samples and that some house-keeping miRNAs may be used as more reliable reference genes for miRNAs in serum. In addition, using the geometric mean of two reference genes could increase the reliability of the normalizers.

## Introduction

Sulfur mustard [bis (2-chloroethyl) sulfide, SM] is a potent vesicant chemical warfare agent which has been extensively used during World War I and more recently against both military and civilian population of Iran during the Iran-Iraq war (1980- 1988). A large number of exposed people still suffer from the long-term effects of SM exposure, especially in their lungs (1).

SM alkylates cell constituents (mainly DNA, but also RNA, proteins and lipid membrane), which ultimately results in cell cycle arrest, apoptosis and/necrosis. In spite of accumulating information regarding pathology of SM injury, there is still an ongoing debate on the exact molecular mechanisms responsible for its acute and chronic effects (2, 3).

MicroRNAs (miRNAs) are a family of endogenously small (20-22 nucleotides) non-coding RNAs that negatively regulate gene expression through translational inhibition or degradation of their target transcripts. A number of important cellular pathways including cell proliferation, differentiation, apoptosis, oxidative stress and inflammation are controlled by these tiny molecules. Their aberrant expression has been associated with some diseases including lung diseases of asthma, chronic obstructive pulmonary disease (COPD) and fibrosis (4-8).

Recent discovery of miRNAs as novel biomarkers in serum and plasma has opened a new field of research in this era. Indeed, circulating miRNAs are stable enough to be detected in serum and plasma of both normal individuals and patients. Moreover, new findings emphasize that any alterations in the serum levels of miRNAs is directly affected by such alterations in original tissues (9). This alteration could reflect the physiological or pathological conditions of the original tissues and also the perturbed molecular pathways responsible for disease initiation and progression (9-13).

Due to the small size of miRNAs, several methods have been employed for their expression analyses including Northern blotting, oligonucleotide microarray, deep-sequencing and quantitative real-time polymerase chain reaction (qRT-PCR). Among these, qRT-PCR has become the method of choice due to its high sensitivity and specificity, as well as its low template requirement (14-16). To achieve reliable and also reproducible qPCR data, non-biological variations resulting from technical inconsistencies should be corrected using an appropriate reference gene (15, 17, 18). This is a critical step in expression analyses, because data normalization with an unsuitable reference gene would lead to biased results (14). Basically, a candidate reference gene should meet certain criteria before being considered as a proper normalizer. These criteria include having the same storage stability, similar extraction and quantification efficiency, comparable length and expression level to the target gene, and most importantly displaying an unchangeable expression level across all samples of the study (17-19). Finding a suitable reference gene is very critical for miRNA studies because i. miRNAs constitute only 0.01% of total RNA mass and this minor fraction is obviously variable across different samples and ii. Their expression level is more susceptible to be altered by technical inconsistencies. Based on previous reports, a single universal reference gene does not exist for miRNA expression analyses in serum (12, 20). Also, the suitability of each candidate should be tested for each study or experimental condition (14, 18, 19, 21). While U6 and 5S rRNA have been widely used as normalizers in miRNA studies, recent findings emphasize the need to identify a more eligible reference gene among miRNA genes (22, 23).

The aim of this study was to find appropriate reference genes in serum samples of patients with SM exposure as a critical pre-requirement for further work on miRNA profiling of these patients. We thus evaluated the suitability of a panel of small RNAs including SNORD38B, SNORD49A, U6, 5S rRNA, miR-423-3p, miR-191, miR-16 and miR-103 in serum samples of SM victims. These candidate reference genes were chosen either based on the literature reviews or by the recommendation of Exiqon (e.g. SNORD38B and SNORD49A) (17, 19, 24, 25).

## Materials and Methods

### Blood sampling

In this case and control study, forty-three male participants including 28 patients and 15 ageand gender-matched healthy controls were included in the study. Patients were selected from a group of veterans who had been simultaneously exposed to high doses of SM during a gas attack in February 1986. The control group comprised of volunteers with no history of SM exposure. Exclusion criteria of both groups were smoking and any history of chronic diseases of lung. Written informed consents were obtained from all participants. This study was approved by the Ethical Committee of Janbazan Medical and Engineering Research Center (JMERC).

Since COPD is one of the late toxic consequences of SM exposure, the Global Initiative for Chronic Obstructive Lung Disease (GOLD) procedure was used to evaluate the severity of pulmonary lesions in these patients. Eight ml of peripheral blood was collected into BD Vacutainer tubes with clot activator and gel (BD, Plymouth, UK). To minimize the inhibitory effects of proteins in serum samples, participants were asked to fast for over 10 hours before sample collection. The serum was harvested by a 20-minutes centrifugation at 2500 g, then aliquoted into RNase-free tubes and stored at −80˚C until use.

### RNA extraction and quantitative polymerase chain reaction

Total RNA was isolated from the samples using the miRNeasy mini kit (Qiagen, Germany) as instructed by the manufacturer. Before adding QIAizol, MS2 RNA (Roche Applied Science, USA) was added to each sample at the final concentration of 1 μg/μl to increase the yield of miRNA extraction. Due to the presence of inhibitors in serum, different volumes of RNA samples (i.e. 0.5, 1, 1.5, 3 and 6 μl) was used for cDNA synthesis in a total volume of 10 μl. Based on our preliminary optimization data, 1.5 μl of each RNA was subjected to qRT-PCR, using a two-step protocol of universal cDNA synthesis and SYBR green master mix kits, along with specific locked nucleic acid (LNA) PCR primer sets (Exiqon, Denmark) on an ABI 7500 real-time PCR machine (Applied Biosystems). Each reaction was performed in duplicate to check technical consistency and the average of their quantification cycle (Cq) was then used in the analyses.

### Data analysis

PCR amplification efficiency was calculated using LinReg 11.0 software (26).

The t test statistic was used for comparison of the distribution of the reference genes expression between the control and patient groups (significance level=0.05). Data was analyzed using SPSS 16.0 software (SPSS Inc, USA). These analyses had to be undertaken before performing other analyses of stability of a candidate gene.

The more recently used algorithms of GeNorm (GenEX software), NormFinder (GenEX software), BestKeeper (27) and comparative delta- Cq were used to analyze the stability of the examined reference genes in all tested samples including cases and controls. Finally, comprehensive gene-stability value was calculated for each gene using the web-based tool, RefFinder (http://www.leonxie.com/referencegene.php). Moreover, geometric mean of 2 reference genes was also considered as a normalizer and its stability was compared with each separate reference gene by geNorm and NormFinder softwares. The geNorm software ranks the tested genes based on their expression stability (M value) and introduces the two most stable reference genes among those tested. The M value describes the mean pairwise variation of a candidate gene compared with all other candidate genes. The stability ranking of each candidate gene was then determined by stepwise exclusion of the gene with the highest M value, followed by recalculation of average expression stability for the remaining genes until the two most stable genes were found.

## Results

### Expression pattern of candidate reference genes in serum

The expression value of 8 candidate reference genes were examined in sera of 9 pooled groups of SM-exposed victims. Among the candidate reference genes, SNORD38B, SNORD49A and U6 had the lowest expression level with a Cq>35 and in some cases undetectable. As a result, they were excluded from further investigation. Among others, a wide spectrum of Cq values ranging from 20.07 to 36.93 was observed. Among all, 5S rRNA was the most abundant candidate with a median Cq of 26.64. The median Cq values of others are shown in table 1.

### Expression of candidate genes in patient and healthy groups

**Fig.1:**
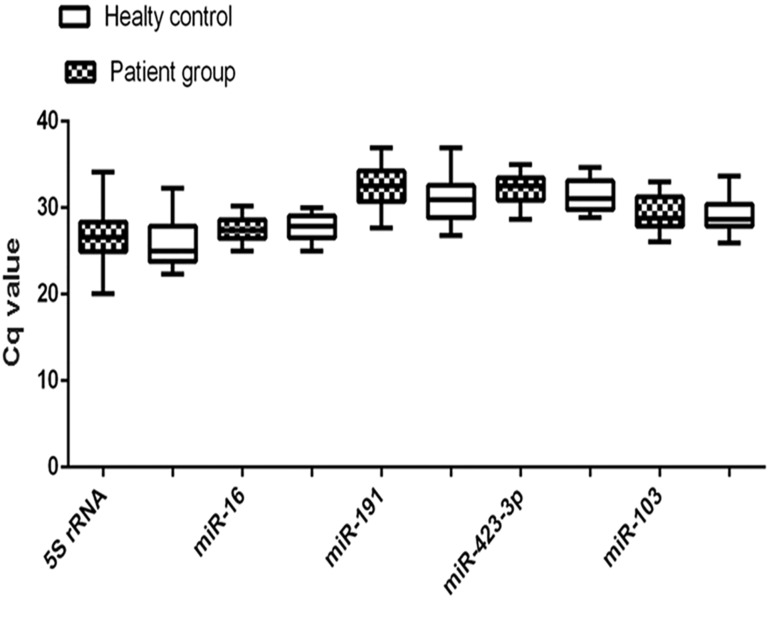
Cq values of candidate genes in tested samples. No differences was found between the control and patient groups (P>0.05). Cq; Cycle of Quantification.

None of the reference genes were affected by the disease state since observed Cq values did not show any significant difference between healthy and patient groups (Fig .1). In addition, the result of t test with P>0.05 for all reference genes indicated that the tested populations of patients and controls have equal variance. This is an essential step before evaluating the stability of reference genes as the algorithms used assume there is no difference in expression pattern of candidate genes between experimental groups.

### Results of expression stability of candidate genes

The results of PCR amplification efficiency for each separate reference gene are presented in table 2.

All the employed software programs assign a relative stability value to each candidate gene which is inversely correlated to its stability. The results of expression stability values are shown in table 3. All the software programs used specified a highest stability for miR-423-3p and a lowest stability for 5S rRNA. The second mostly stable reference gene was miR-103 based on Normfinder and delta Cq, and miR-16 based on Genorm and BestKeeper analyses. This discrepancy could be attributed to the different algorithms used by these softwares. Therefore, to reach a conclusion, comprehensive gene-stability value was also calculated for each gene (Table 3).

**Table 1:** Descriptive statistical values of Cycle of quantification of reference genes in 43 tested samples


Rank	Gene	Min	Max	Average	Median	SD

1	miR-423-3p	28.67	35	31.93	31.9	1.83
2	miR-16	24.98	30.23	27.63	27.44	1.46
3	miR-103	25.95	33.7	29.27	28.65	2.01
4	miR-191	26.83	36.93	31.94	32.07	2.6
5	5S rRNA	20.07	34.15	26.64	25.97	3.21


**Table 2:** PCR efficiency of candidate reference genes


Rank	Gene	Accession	PCR efficiency

1	miR- 4 2 3 - 3 p	MIMAT0001340	0.920
2	miR- 1 6	MIMAT0000069	0.850
3	miR- 1 0 3	MIMAT0000101	0.910
4	miR-191	MIMAT0000440	0.870
5	5 S rRNA	V00589	0.880
6	U6	NR_002752	not detected
7	SNORD38B	NR_001457	not detected
8	SNORD49A	NR_002744	not detected


PCR; Polymerase chain reaction.

**Table 3:** Expression stability of reference genes across 43 tested samples. miRNAs are ranked based on their comprehensive gene-stability value


Rank	Gene	NormFinder	geNorm	BesteKeper	Delta Cq	Comprehensive

1	miR-423-3p	1.11	2	1.53	2.27	1.19
2	miR-16	1.85	2	1.19	2.59	1.86
3	miR-103	1.71	2.11	1.68	2.54	2.45
4	miR-191	1.85	2.31	2.1	2.61	3.72
5	5S rRNA	2.255	2.6	2.49	3.04	5


Cq; Cycle of quantification.

**Fig.2:**
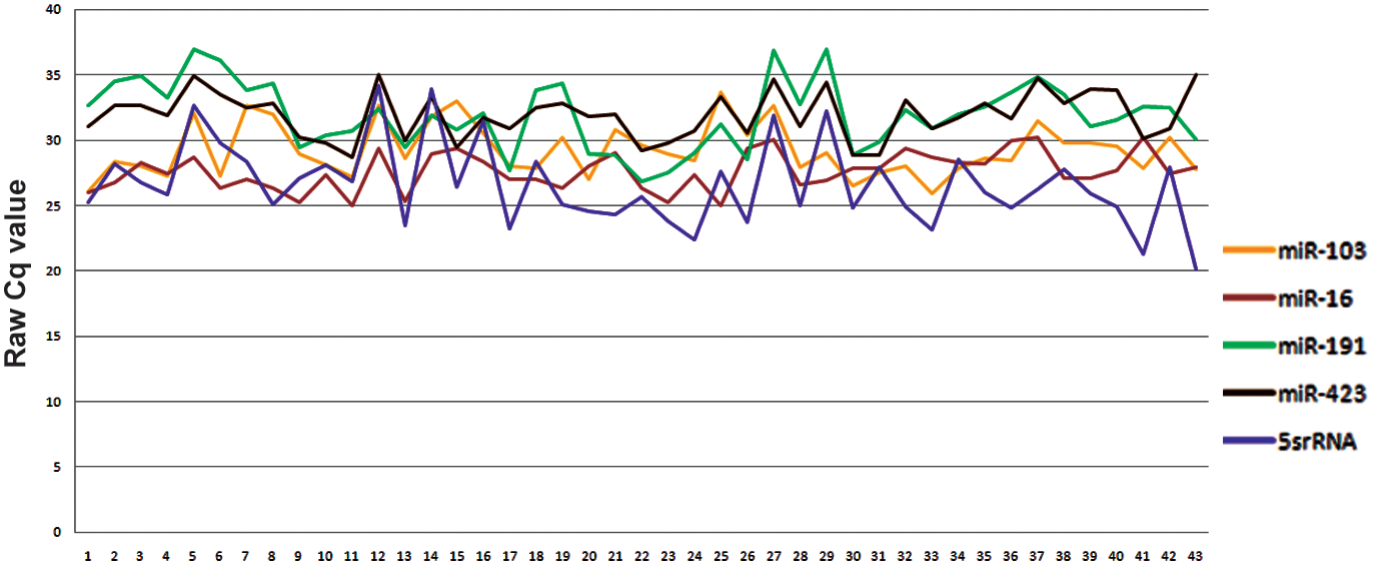
The variation in expression level of 5 reference genes investigated in serum samples of 43 participants in this study. The expression level was estimated using qPCR (based on duplicate readings). qPCR; Quantification polymerase chain reaction.

To visualize expression variation of reference genes in these samples, raw Cq values were plotted. As shown in figure 2, the highest variation belonged to 5S rRNA and miR-191, therefore being the two least stable reference genes (Fig .2).

**Fig.3:**
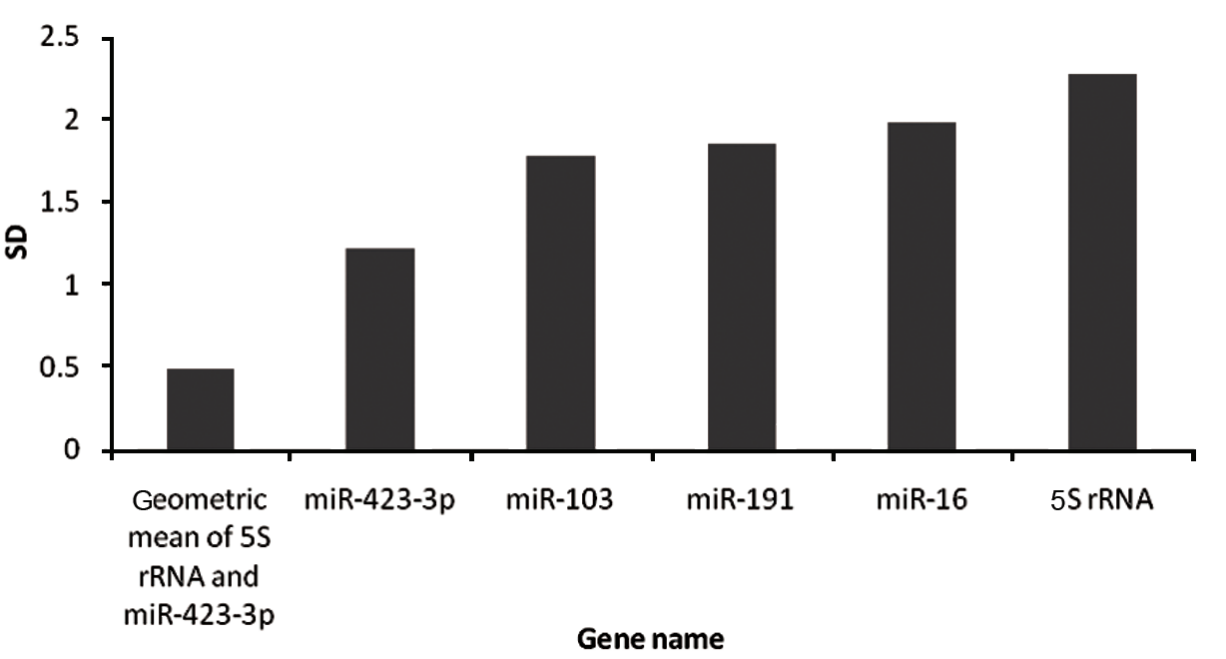
Normfinder analysis of 5S rRNA after applying geometric mean as a separate normalizer. The expression stability of each candidate is shown by SD in NormFinder. The lower value of SD represents higher gene stability. SD; Standard deviation.

**Fig.4:**
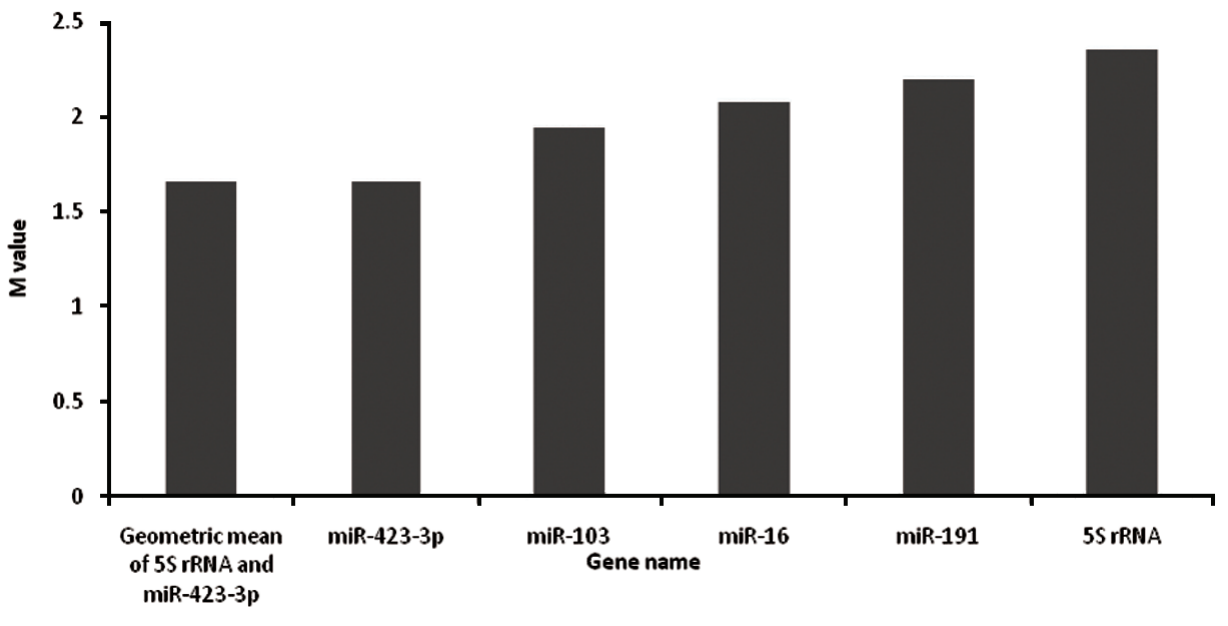
GeNorm analysis of 5S rRNA after applying geometric mean as a separate normalizer.

Finally, geometric mean of two reference genes were applied as a separate normalizer, and its stability was compared with other single candidate genes using Genorm and Normfinder programs. The data revealed that the stability of the geometric mean normalizer is significantly higher than each candidate, even when the least stable reference gene, 5S rRNA, was included (Figes.3, 4). As shown in figure 3, standard deviation (SD) of 5S rRNA was decreased from 2.3 to 0.51 after adding its geometric mean with miR-423-3p. Similar observation was made after adding obtaining the geometric mean of 5S rRNA with that of miR-423 using geNorm software (Fig .4).

## Discussion

While a large number of studies have addressed the assessment of reference genes in mRNA qRT-PCR studies, evaluation of eligible reference genes for miRNA qRT-PCR studies is still in its infancy. This new field of study needs to be well-established especially in the case of serum samples because the stability of commonly used reference genes is strongly affected by the presence of large amounts of RNase in such samples.

Several different strategies can be used for normalization of qPCR data. These include normalization to sample size or volume, total RNA and also to an internal reference gene. The use of reference gene is the most universal and accurate method of normalization in qRT-PCR studies (28), especially when only a few genes are under investigation. Nuclear and nucleolar RNAs are commonly used as normalizers in expression studies of miRNAs. Recently, however, a number of studies have found biases in their suitability, calling into question their reliability for accurately quantifying miRNAs (22, 23, 29). For this reason, the suitability of 8 candidate genes from both categories was evaluated in this study. Of these, 4 belong to nuclear, nucleolar and ribosomal non-coding RNAs and the remaining from the miRNA category.

The reason for doing the present case-control study of SM-exposed patients was for our future follow-up project on miRNA profiling in the same serum samples. As a pre-requirement, it is highly recommended to identify a reliable reference gene with an unchangeable expression level across all samples of the study, including patients and controls. Indeed, the expression level of the reference genes should not be affected by disease state. Panels of miRNAs have been suggested as reliable reference genes in various physiological contexts, however, this needs to be done in SM-exposed people before doing miRNA quantification. Herein, stability of 8 candidate reference genes were just evaluated in serum samples of SM-exposed veterans. Therefore, it remains to be determined if our findings (the reference genes introduced as reliable candidates) can be applied to other diseases. Lack of significant difference in expression level of tested candidates between cases and controls means that they are not influenced by the physiological state of this disease and can be considered for further analysis.

Altogether, we conclude that candidates of miRNA category have a higher stability in serum, and that only 5S rRNA from the non-coding RNA category is suitable as a reference gene. This is because, circulating miRNAs are released in exosomal vesicles which make them more stable against RNase degradation compared with small nucleolar RNAs (SNORD38B, SNORD49A) and small nuclear RNAs (U6).

Although miR-423-3p was identified as the most stable reference gene, the higher expression level of miR-103 and miR-16 make them better candidates than miR-423-3p.

Appropriateness of miRNAs as endogenous control genes has been proposed in other studies. Song et al. (23) reported the suitability of miR-16 and miR-93 in serum samples of gastric cancer. They also showed that U6 had the lowest expression level in serum samples. Lim et al. (22) showed U6 and 5S rRNA are not reliable miRNA reference genes in neuronal differentiation and they proposed the combination of three miRNAs including miR-103 for accurate expression normalization. Peltier and Latham (19) examined the stability of 16 candidate reference genes in 5 pair of distinct tumor/normal adjacent tissues. Their finding showed miR-103 and miR-191 had highly consistent expression level compared to most commonly used reference genes including U6 and 5S rRNA.

In general, few studies have evaluated the suitability of reference genes in serum samples. Song et al. (23) introduced miR-16 as one of the two most stable reference genes in sera of patients with gastric cancer. Although several reports have introduced miR-16 as one of the most stable reference gene in miRNAs studies, other reports have addressed its aberrant expression in malignancies (30-32). In addition, increased expression level of miR-16 during haemolysis has been previously reported. Therefore, as miR-16 is the second consistent reference gene in these samples, great caution should be taken in serum harvesting to avoid red blood cell haemolysis (33). Zheng et al. (20) evaluated stability of 13 candidate reference genes in sera samples of colorectal adenocarcinoma and introduced miR-191 as the most stably expressed reference gene in these samples.

We also applied geometric mean of two reference genes as a normalization factor, finding that applying more than one reference gene would result in more stability even in the case of the least stable reference gene. This is an applicable finding, especially when researchers are unable to choose a suitable reference gene from a panel of candidates. To reduce technical variations, we therefore suggest normalizing data using geometric mean of 2 reference genes.

## Conclusion

We demonstrate that miR-423-3p, miR-16, and miR-103 are the most stable reference genes in serum of sulfur mustard victims. Also, using two reference genes is recommended especially when there is no choice to select a stable normalizer.
